# Co-Invasion of Congeneric Invasive Plants Adopts Different Strategies Depending on Their Origins

**DOI:** 10.3390/plants13131807

**Published:** 2024-06-30

**Authors:** Yujun Guo, Meini Shao, Ping Guan, Mengyang Yu, Lin Geng, Ying Gao, Lin Meng, Bo Qu

**Affiliations:** 1 College of Bioscience and Biotechnology, Shenyang Agricultural University, Shenyang 110866, China; thanks4u1228@163.com (Y.G.); smn@syau.edu.cn (M.S.); 2002500027@syau.edu.cn (P.G.); 2 Liaoning Key Laboratory for Biological Invasions and Global Changes, Shenyang Agricultural University, Shenyang 110866, China; 3 Yixian Water Conservancy Affairs Service Center, Jinzhou 121100, China; a10881025@126.com (M.Y.); 13188121099@163.com (L.G.); 18840124411@163.com (Y.G.); 18941679977@163.com (L.M.)

**Keywords:** co-invasion, origin difference, congeneric invasive plants, Darwin’s naturalisation conundrum

## Abstract

Plant communities may be co-invaded by invasive plants, sometimes even by congeneric invasive plants (CIPs). Despite the growing understanding of co-invasion in the environment, little is known about how CIP interactions and mechanisms regulate co-invasion. Darwin’s naturalisation conundrum predicts that the coexistence of closely related species is difficult due to their structural and behavioural similarities. Nevertheless, communities containing closely related species are more susceptible to being invaded because close relatives may favour similar environments; therefore, this hypothesis should be followed in the co-invasion of CIPs. To explore whether the phylogenetic relatedness and origins of invasive species to CIPs can promote or hinder co-invasion, we conducted a controlled interaction and soil-legacy greenhouse experiment to quantify the growth response of invasive plants and their congeners. We consistently found that CIPs of identical origin were more likely to co-invade compared to CIPs of distinct origins. CIPs of distinct origins exhibited an antagonistic effect on co-invasion by allelopathy. Invasive plant-conditioned soil was more conducive to the growth of CIPs of identical origin than CIPs of distinct origins. Our results revealed the different effects of invader–invader phylogenetic relatedness on co-invader success and impact, suggesting the operation of different mechanisms across co-invasion.

## 1. Introduction

Increasing anthropogenic activities, such as transportation, agriculture, and global trade, have significantly facilitated the invasion of species into suitable habitats [1,2]. The communities may be invaded by two or more invasive species, a phenomenon referred to as co-invasion [3]. By 2050, invasive species are projected to increase by 36%, implying an increased chance of co-invasion [4]. For instance, they may interact with one another or alter the rhizosphere microbial community, which influences the likelihood that more invasive plants will invade, ultimately impacting the ecosystem.

The presence of invasive species will unavoidably influence their invasive neighbours and subsequent invasive plants (either accelerating or decelerating). It has become an urgent ecological problem to predict which invasive plants will co-invade, the effect of invasive plants on subsequent invasive plants in the community, and how to effectively prevent, manage, and control co-invasion.

The emergence of phylogenetic biology has facilitated the use of phylogenetic relatedness and evolutionary history in investigating ecological problems [5,6,7]. Darwin [8] first proposed that phylogenetic relatedness plays a crucial role in plant competition. Alien species are less likely to invade communities containing their close relatives because of niche overlap and similar resource needs, resulting in strong competition, known as ‘Darwin’s naturalisation hypothesis’ (DNH) [9]. In addition, invasive plants occupy similar environments, leading to an early adaptation process for subsequently closely related plants. Environmental filtering further enhances the susceptibility of closely related species to invasion, referred to as the ‘Pre-adaptation hypothesis’ (PAH) [10]. The two hypotheses, collectively known as ‘Darwin’s naturalisation conundrum (DNC)’, describe the potential contrasting influences of phylogenetic relatedness on invasion [11,12,13]. Studies have found that biotic similarity among invasive plants increases invasiveness [14,15]. Biologic interactions and environmental filtering have both been shown to be potential determinants of biological invasion [16]. However, the mechanisms underlying the advantages of co-invasion of closely related species over their respective invasion remain unclear.

Interactions between plants are usual and can have direct effect (space occupancy and competition for shared resources) [17,18] or indirect effect (through changes in plant surroundings, biodiversity, and ultimately ecosystem function) [19]. Resource competition can be divided into two dimensions: ‘competitive effect’ (the ability of species to take up high resources) [20,21,22] and ‘competitive response’ (the ability of species to tolerate low resources) [23,24,25,26]. Allelopathy and soil microorganism regulation can have an indirect effect on the growth of other invasive species [27,28]. The allelopathy of invasive plants, an interference mechanism, is pervasive [29]. Co-invading species can synergistically affect the abundance of soil bacterial community [30], and invasive plants can build an environment more conducive to competitive ability and thus the invasiveness of subsequent invasive plants (Invasional meltdown hypothesis) [31]. Noteworthy, invasive plant-conditioned soil was not likely to directly affect competition intensity from subsequent plants; instead, the similarity in soil microbial communities indirectly affected subsequent invasive plants [32]. As a result, we believe that the traditional phylogenetic method or the functional similarity approach to studying the effect of phylogenetic relatedness on co-invasion may overlook the contribution of some specific conditions (for example, differences in species origin or direct and indirect effects among plants).

Recent studies have revealed that invasive populations in invasive areas can evolve to have more significant ecophysiological advantages than native populations through altering biomass allocation, which is conducive to resource competition and vigorous growth [33,34,35]. However, it is unclear whether co-invasion in invasive areas is affected by their origin (which could be attributed to allelopathic interference) [36]. In recent years, co-invasion research has relied primarily on observational data, with limited experimental data [37,38,39,40]. Simultaneously, these studies have not directly examined the intensity of interaction among species from the perspective of infrageneric relationships. Such data are crucial for understanding the consequences and mechanisms of co-invasion in a specific ecosystem. In this study, we identify the potential linkage between competition for shared resources and allelopathic interference with congeneric invasive plants (CIPs) that co-invade, and specifically test whether phylogenetic relatedness and the origin of species affect co-invasion of CIPs. Furthermore, we addressed potential mechanisms of co-invasion of CIPs through plant interaction and allelopathic legacy experiments. We investigated the following key questions: (1) Does significant interspecific competition exist among CIPs? If interspecific interaction is more significant than intraspecific interaction, we believe they will fail to co-invade or vice versa. Are their divergent outcomes of interactions depending on their origins? If so, (2) How do nutrition and allelopathy affect the co-invasion of CIPs, and (3) How do CIPs adjust the biomass allocation strategy when they co-invade?

## 2. Materials and Methods

### 2.1. Study Location and Species

The common garden experiment was carried out in a plastic canopy (8 m × 60 m) in the Teaching and Research Base of Shenyang Agricultural University (41°49′48.57″ N, 123°33′42.65″ E) from 2022 to 2023.

**Figure 1:**
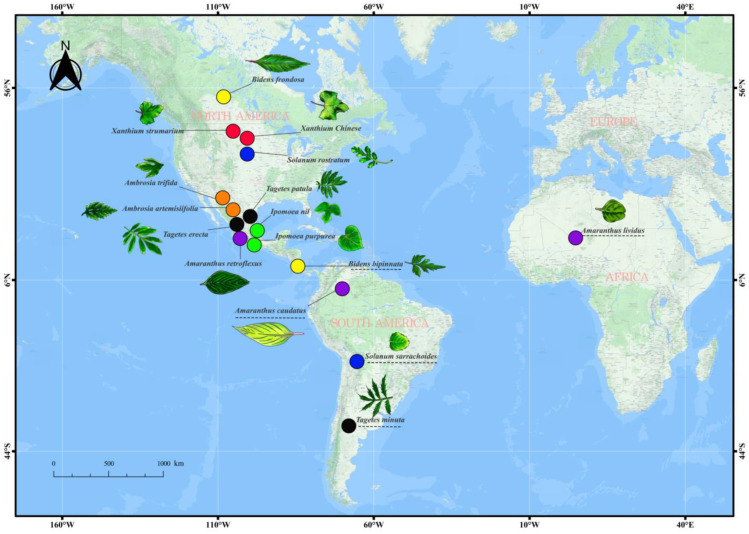
The picture shows the origins of species in this experiment. The same colour represents the same genus. The underscore represents distinct origins of the same genus.

We consider two species of the same genus as a group and then subgroup them according to geographical origin differences. If both species are native to the same continent, we consider them of an identical origin (IO). For example, *Ambrosia trifida* and *Ambrosia artemisiifolia* are native and widespread in North America. On the contrary, *Solanum rostratum* (North America) and *Solanum sarrachoides* (South America) are native to distinct continents. Therefore, we consider them as plants of distinct origins (DOs) (Figure 1, Table S1). All seeds were collected from the field. Additionally, to account for possible regional variations, invasive species belonging to the same genus were collected from distinct geographical regions (thus prioritizing plants originating from different provinces or cities when collecting CIPs). Based on the ‘Chinese Alien Plant List’ and the Chinese Biobank (https://species.sciencereading.cn, accessed on 22 June 2021), we identified the experimental species as alien species [41]. Among them, we selected 16 alien species (7 families), all successfully established in China.

### 2.2. Experimental Set-Up

In April 2022, seeds for all plants were immersed in a 1% sodium hypochlorite solution for 20 min and then rinsed five times with distilled water. Subsequently, they were germinated in a light incubator or at room temperature under conditions of light/dark 25 °C/20 °C or light/dark 30 °C/25 °C, respectively. Once most of the seeds had germinated (with the radicle length equal to the seed length as the standard), we transferred them to hole plates for seedling cultivation. Seedlings that reached the ‘three-leaf and one-core stage’ and exhibited robust growth were transplanted into pots (27 cm × 17 cm). We sifted the soil in the field using a 1 cm screen to remove plant material and large stones while incorporating soil, substrate, and vermiculite into an experimental soil mixture in a 3:1:1 ratio.

**Figure 2:**
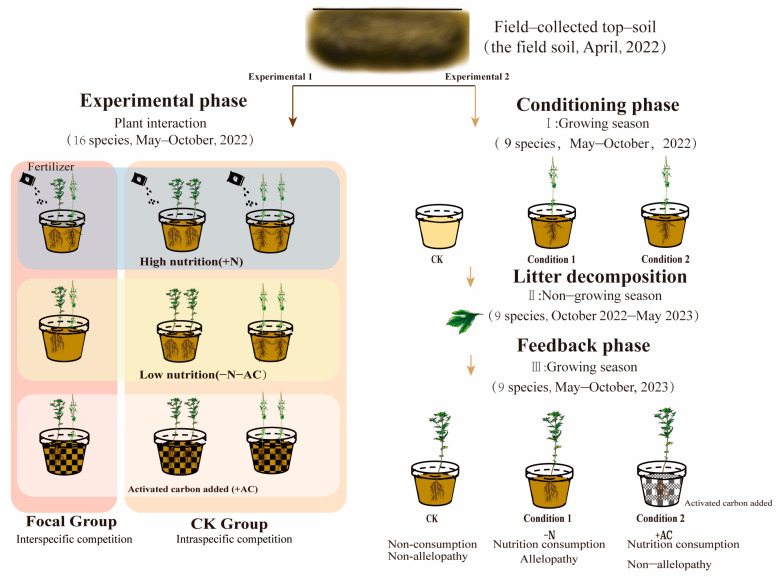
The design of the two-phase experiment in this study.

**Experiment 1** addressed the effects of origin differences, nutrition, and direct allelopathy on co-invasion of CIPs. We selected two plants per pot based on the combination requirements (Figure 2, Table S2). We planted 16 combinations of interactions: 11 combinations of interspecific interactions and 16 combinations of intraspecies interactions with a seedling spacing of 8 cm. Each group had five replicates of interspecific interactions, and one target species was taken from each pot to measure the experimental data. Intraspecific interaction was repeated three times in each group, and two target plants were taken from each pot to measure experimental data. We planted a total of 618 plants in 309 pots in the experiment. If seedlings died within two weeks of planting, they were replaced with new ones. After two weeks of planting, we conducted experimental treatments in three groups: high-nutrition group (+N), low-nutrition group (−N−AC), and allelopathic treatment group (+AC). We applied a water-soluble fertilizer (N:P:K:S = 24:12:14:4) to the soil in plants in the +N group. A 10 g solution dissolved in 4 L of water was administered to the roots monthly, totalling four applications. −N−AC group had no compound fertilizer and activated carbon. The +AC group contained additional activated carbon and a soil ratio of 20 mL/L. Pre-experiment and the literature proved that adding activated carbon had no significant effect on plant growth [42]. We irrigated the plants daily from 17:00 to 18:00 to maintain optimal soil moisture levels. Once planted, we sprayed all plants with pesticides to minimize pest and disease interference. We moved the pots randomly every two weeks throughout the experiment to prevent the influence of marginal effect on the experiment; 16 weeks after transplanting, we measured the plant phenotype (including height, coverage, and basal diameter). We used the LI-3000 leaf area meter (LI-Cor, Lincoln, NE, USA) to measure the collected leaves’ single leaf area (LA). Photosynthesis was measured using an LI-6400XT portable photosynthesis system (LI-Cor, Lincoln, NE, USA). We harvested plants at the ground level to obtain aboveground biomass and then separated the stems, leaves, and seeds for individual collection. Subsequently, we dried these plant parts in an oven at 80 °C until a constant weight was reached before weighing. Following this, we used high-pressure water to wash away the soil surrounding the roots of both species in each pot. The roots were carefully separated as much as possible in the water and dried in an oven until a constant weight was reached. We believe that losing a tiny part of fibrous root biomass during the separation process will not significantly affect the experimental results. As outlined by Poorter [43], we derived several parameters from our measured data: leaf mass fraction (LMF), root mass fraction (RMF), stem mass fraction (support organ mass fraction, SOMF), seed mass fraction (SMF), and specific leaf area (SLA).

**Experiment 2** addressed the effects of soil legacy (indirect allelopathy) and nutrition depletion on subsequent CIPs. During the soil treatment stage from May to October 2022, seedlings were carefully selected from each pot and cultivated throughout the growing season based on specific requirements (Figure 2, Table S3). We conducted experimental treatments under three conditions: the control group (no plants in the first year and no activated carbon added in the second year, designated as CK), condition 1 (plants in the first year and no activated carbon added in the second year, designated as −N), and condition 2 (plants in the first year and activated carbon added in the second year, designated as +AC). We planted the required number of each plant (9 species) and replicated each group eight times to give a total of 264 pots containing 176 plants. In order to replicate the natural growth conditions of plants in the field, we did not immediately remove plants; leaves were allowed to decompose naturally in the soil. We did this because of uncertainty about whether root exudate metabolites and leaf litter decomposition would have varying effects on the subsequent plants. Consequently, we removed the entire plant before conducting the soil feedback experiment in the second year. In the soil feedback stage, 264 pots and 264 plants (9 species) were planted under three conditions. Seedlings were selected per pot and cultivated throughout the growing season from May to October 2023. After 16 weeks of transplanting, we harvested the above-ground biomass by cutting the plants at ground level, and we harvested the below-ground biomass by washing the soil around the roots using high-ressure water. All biomass was dried until it reached a constant weight before being weighed.

### 2.3. Statistical Analyses

According to Goldberg’s definition, the competitive effect is the capacity to restrict the growth or reproduction of neighbouring species, and the competitive response is the ability to withstand the negative effects of neighbouring species [44,45]. To investigate the universality of phylogenetic relatedness between CIPs and avoid pseudoreplication, we conducted measurements of indices under consistent disturbance factors and environmental conditions. On the same day, we measured physiological indices of the same species, such as plant height, photosynthesis, and so on. The resulting data were logarithmically transformed for analysis purposes. We used a standard index and calculated the log response ratio (ln RR) [46]:

(1) $$ln\;RR=ln(\frac{X_{focal}}{X_{CK}}),$$

*X_focal_* represents the individual when the target species competes with neighbouring CIPs in interspecific interaction and *X_CK_* denotes the average total measurement of the target species in intraspecific interactions. The independent variables in this study were the experimental treatment conditions and the dependent variable was the response coefficient of the measurement index ln RR. This approach allowed for comparing competitive abilities among CIPs, regardless of plant size. For the convenience of analysis, we selected greater total biomass response coefficient values as predominant species (PS) in −N−AC and inferior species (IS) with lower values (Table S2). In the analysis, we examined the significance of plant origin on interaction and allelopathic legacy. We used a continuous index of allelopathy to test for the allelopathic legacy of CIPs in monocultures as follows [47]:

(2) $$\text{Allelopathic legacy in monocultures = ln}\frac{+AC}{CK}-\ln\frac{-N}{CK},$$

*+AC* represents the individual when the target species is in +AC, −*N* represents the individual when the target species is in −N, and *CK* denotes the average total measurement of the target species in CK. Negative and positive values indicate a negative and positive effect of allelopathic legacy of invasive plants, respectively.

Data were expressed as mean ± standard error (SE) and analysed using variance analysis followed by the Bonferroni test. All data were analysed using Prism 10.1.1 and pathway analysis was performed with R 4.3.0 software to evaluate the effect size and path of nutrition and allelopathy on plant competition.

## 3. Results

### 3.1. Interactive Effects of Species Origin Differences, Nutrition, and Direct Allelopathy

**Figure 3:**
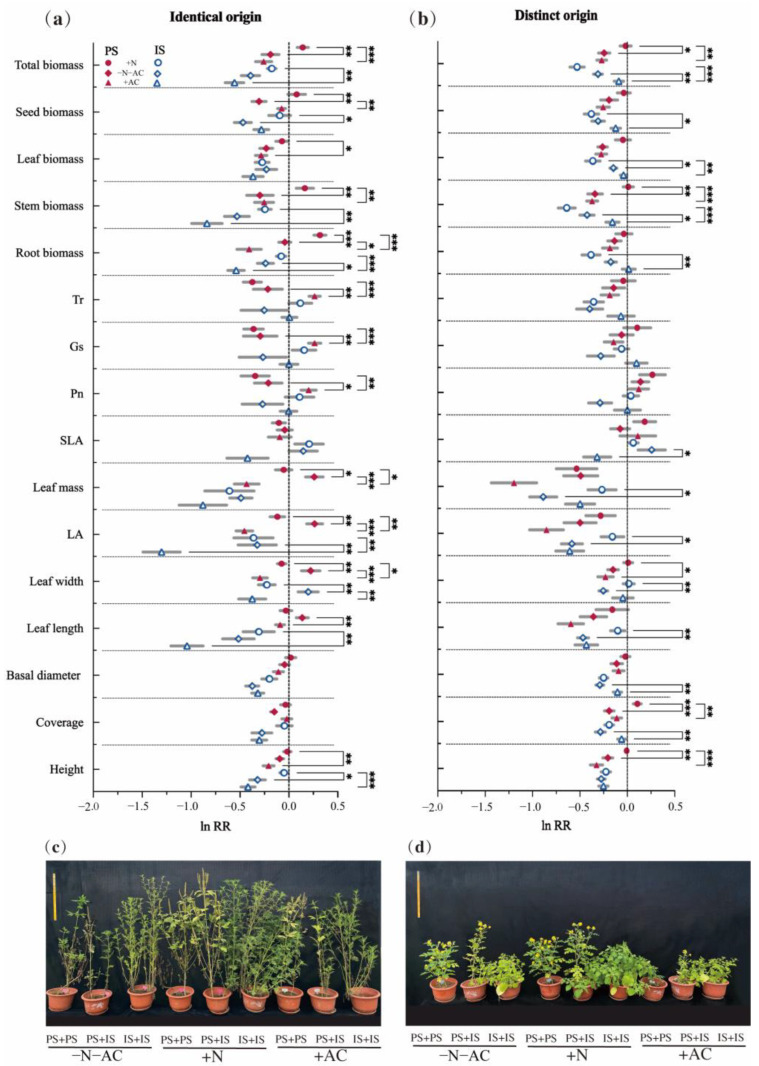
The relative change in (**a**,**c**) identical origin (IO) and (**b**,**d**) distinct origin (DO) species in response to nutrition and allelopathy interactions. Grey lines represent the SE (standard error). Values are mean ± SE (relative changes). *, *p* ≤ 0.05; **, *p* ≤ 0.01; ***, *p* ≤ 0.001. Values of *p* indicate significant differences. Values of *p* > 0.05 are not presented for clarity. PS: predominant species, IS: inferior species, Tr: transpiration rate, Gs: stomatal conductance, Pn: net photosynthetic rate, SLA: specific leaf area, LA: single leaf area.

**Figure 4:**
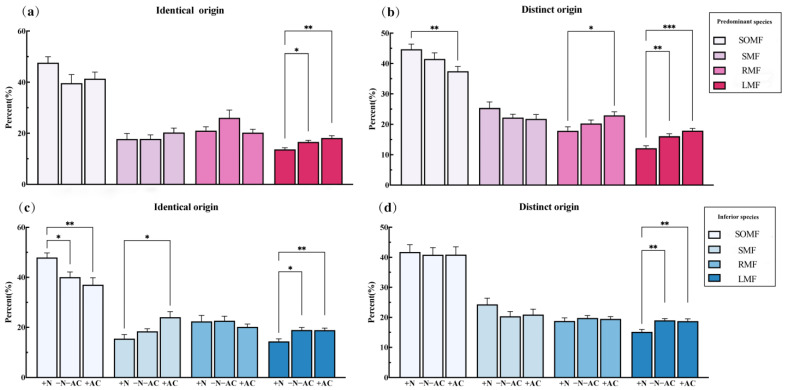
The relative changes in the proportion of biomass in (**a**,**c**) identical origin and (**b**,**d**) distinct origin species. SOMF: support organ mass fraction, SMF: seed mass fraction, RMF: root mass fraction, LMF: leaf mass fraction. *, *p* ≤ 0.05; **, *p* ≤ 0.01; ***, *p* ≤ 0.001. Values of *p* indicate significant differences. Values of *p* > 0.05 are not presented for clarity.

Under high-nutrition conditions (+N), the total biomass of PS was consistently higher than that of the low-nutrition group (−N−AC). This difference was significant for both identical origin (IO, mean difference (md) = 0.33, *p* = 0.003) and different origin (DO, md = 0.226, *p* = 0.03) species. At the same time, other biomass of PS of IO and DOs showed a positive increase, with significant increases in seed biomass (IO, md = 0.385, *p* = 0.002; DOs, md = 0.154, *p* = 0.53), stem biomass (IO, md = 0.4595, *p* = 0.007; DOs, md = 0.351, *p* = 0.001), and root biomass (IO, md = 0.359, *p <* 0.001; DOs, md = 0.098, *p* > 0.05). However, the total biomass of IS is not always higher than that of −N−AC. This difference was opposite for identical origin (IO, md = 0.217, *p* = 0.14) and different origin (DO, md = −0.22, *p* = 0.05) species (Figure 3). For the PS of IO, the leaf biomass was lower than that of the control group, while other biomass showed larger values (ln RR > 0). Only seed biomass (md = 0.376, *p* = 0.04) and SOMF (md = 7.817, *p* = 0.03) of IS of IO demonstrated significant increases (Figure 3 and Figure 4c). Under low-nutrition (−N−AC), the total biomass of all species was lower compared to the control group (ln RR < 0). Regardless of the origin of the species, there was a decrease in SOMF of PS and an increase in RMF with decreasing nutrition levels (Figure 4a,b). Under the condition without allelopathy (+AC), the total biomass of IS of DOs significantly increased (md = 0.22, *p* = 0.006), and the total biomass of other species was lower than that under the condition with allelopathy (−N−AC). Changes in the biomass of the root, stem, leaf, and seeds of PS and IS of DOs were the same as the total biomass (Figure 3b). In particular, after excluding allelochemicals from consideration, the effects of nutrition on all species were highly significant (PS of IO, md = 0.4, *p* < 0.001; IS of IO, md = 0.38, *p* = 0.003; PS of DOs, md = 0.251, *p* = 0.006; IS of DOs, md = 0.44, *p* < 0.001, Figure 3). Under the low-nutrition and without allelopathy conditions, the LMF of PS and IS was significantly higher than that of high-nutrition (−N−AC/+AC: PS of IO, md = 2.935/4.445, *p* = 0.01/0.002; IS of IO, md = 4.578/4.495, *p* = 0.01/0.006; PS of DOs, md = 3.916/5.757, *p* = 0.004/<0.001; IS of DOs, md = 3.842/3.571, *p* = 0.002/0.01, Figure 4). After allelochemicals were excluded, the change in seed biomass of PS and IS of IO was opposite to that of total biomass, and the seed biomass of PS showed a significant increase (md = 0.23, *p* = 0.009, Figure 3a).

### 3.2. Growth Traits Response to Nutrition and Allelopathy Changes

**Figure 5:**
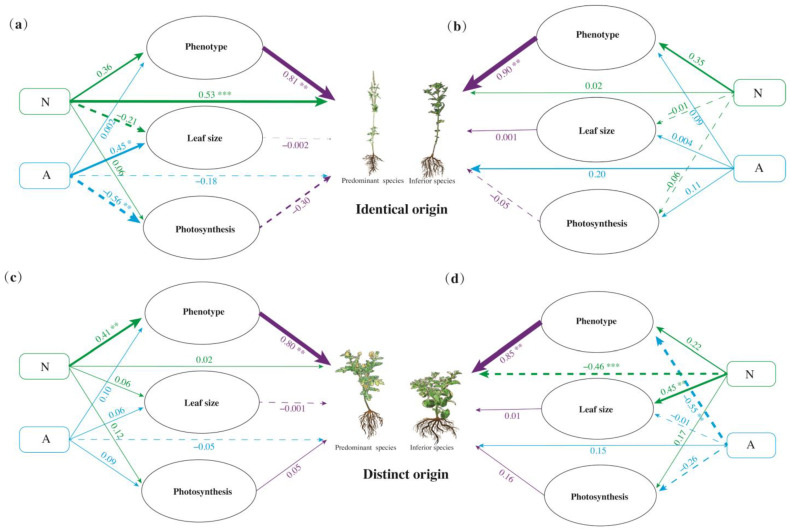
Structural equation model (SEM) of the effects of nutrition (N) and allelopathy (A) interactions on the fitness of the predominant species (**a**,**c**) and inferior species (**b**,**d**). Solid and dotted lines indicate positive and negative correlations, respectively. Green, blue, and purple lines illustrate the nitrogen, allelopathy, and plant growth traits path diagram related to plant fitness, respectively. N: Nitrogen, A: Allelopathy. *, *p* ≤ 0.05; **, *p* ≤ 0.01; ***, *p* ≤ 0.001. Values of *p* indicate significant differences. Values of *p* > 0.05 are not presented for clarity.

In general, nutrition had a positive effect on co-invasion by plant ‘Phenotypes’ (PS of IO = 0.2916; IS of IO = 0.315; PS of DOs = 0.328; IS of DOs = 0.2244, Figure 5). However, nutrition had significant and negative effects on the co-invasion of IS of DOs (direct effect: −0.46, *p* < 0.001), while they exhibited significant and positive effects on the co-invasion of PS of IO (direct effect: 0.53, *p* < 0.001). These findings were contrary to those observed for ‘Leaf size’ (PS of IO = −0.21, *p* > 0.05; IS of DOs = 0.45, *p* < 0.01) (Figure 5a,d). Allelopathy directly generated a negative influence on PS (direct effect: IO = −0.18, DO = −0.05) and a positive influence on IS (direct effect: IO = 0.20, DO = 0.15). Nutrition had no significant effects on ‘Photosynthesis’; however, allelopathy had a significant negative effect (direct effect: −0.56, *p* < 0.01) on ‘Photosynthesis’ of PS of IO and a positive effect (direct effect: 0.11, *p* > 0.01) on IS. Notably, the allelopathy’s effects on PS and IS of DOs were opposite (Figure 5c,d). Allelopathy positively influenced the ‘Phenotype’, ‘Leaf size’, and ‘Photosynthesis’ of IS of IO. In contrast, the opposite influenced IS of DOs and significantly and negatively influenced ‘Phenotype’ (direct effect: −0.55, *p* < 0.01) (Figure 5b,d).

### 3.3. Effects of Invasive Plants on Subsequent CIPs

**Figure 6:**
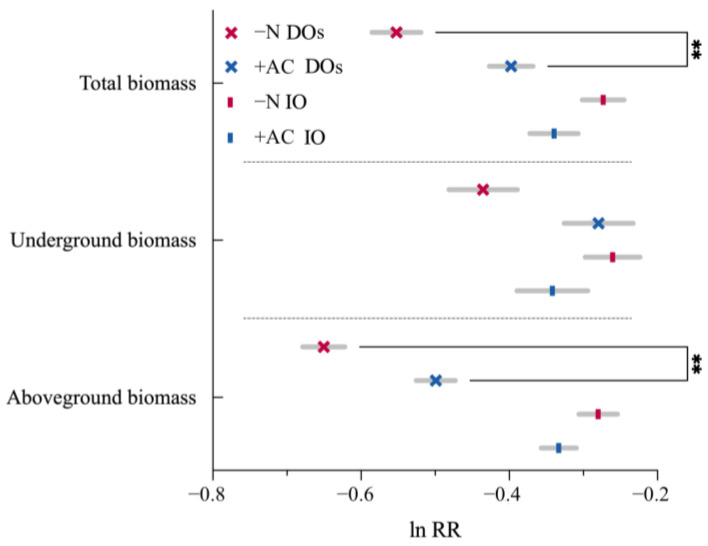
Soil treatments with congeneric invaders. DOs (×): CIPs native to distinct origins; IO (|): CIPs native to identical origins. **, *p* ≤ 0.01. Values of *p* indicate significant differences. Values of *p* > 0.05 are not presented for clarity.

Generally, the total biomass in IO and DOs varied because of the soil-egacy effect (Figure 6). The soil conditioned by invasive plants significantly and indirectly generated a negative influence on subsequent CIPs of DOs (Allelopathic legacy in monocultures: md = −0.151, *p* = 0.001) while promoting the invasion of IO (Allelopathic legacy in monocultures: md = 0.053, *p* > 0.05). Furthermore, the allelopathic legacy had a consistent impact on both above-and below-ground biomass.

## 4. Discussion

The origin of invasive species can have important ecological consequences for co-invasion of CIPs [48,49,50]. At the same time, nutrition and allelopathy also play a crucial role in facilitating the co-invasion of CIPs of IO [51,52,53,54]. Specifically, under sufficient nutrition level, the total biomass of IO species has no significant difference between inter- and intra-specific interactions (Figure 3a). However, the total biomass of DO species shows a significant difference (Figure 3b). As a result, we believe CIPs of IO are more likely to co-invade a habitat than CIPs of DOs. In this experiment, CIPs co-invade at a high nutrition level, prioritising reducing stem biomass allocation while increasing the allocation to root and leaf biomass (Figure 4 and Figure 5). The co-occurring invasive–invasive plant strategy is not the same as the invasive–native plant strategy [55,56]. Invasive plants must adjust the allocation (higher or lower) of seed biomass according to their neighbouring collaborators or rivals for survival [57]. Following the exclusion of allelochemicals, PS and IS of IO will allocate a reduced amount of root biomass and invest more biomass into seed production, aiming to compete for additional nutrition and ensure stable reproduction (Figure 3 and Figure 4a,c). Notably, when CIPs of IO co-invade, PS of IO gain a competitive advantage, they exhibit significant reductions in single leaf mass, leaf area, leaf length, leaf width, and specific leaf area (SLA). In contrast, PS of DOs have the opposite strategy. The single-leaf growth pattern is in alignment with photosynthesis. These findings suggest that CIPs employ distinct nitrogen allocation strategies to regulate leaf construction costs during co-invasion [58]. The stems serve as the primary vegetative organs responsible for plant transportation and support, playing a crucial role in protecting against external environmental factors [59]. IS reduce biomass allocation to their stems, significantly reduce plant height, and adjust the cost of transport, thereby ensuring the stable reproduction of species. When CIPs of DOs co-invade, PS of DOs compete fiercely with IS. PS of DOs allocate more resources to support organ biomass and reduce the proportion of root and leaf biomass allocation to ensure greater height and coverage. Additionally, PS of DOs invest more resources into seed production to secure a reproductive advantage for their species. IS of DOs prefer allocating less leaf biomass while significantly increasing a single leaf’s area, mass, length, and width. Preferentially, IS of DOs allocate more biomass to seed biomass to ensure stable species reproduction. Notably, the strategy of IS of DOs is diametrically opposed to that of PS of DOs. The nitrogen allocation strategy of PS of IOs is active, while that of IS of DOs is passive. Thus, this study found that actively reducing leaf construction costs can promote species abundance and accelerate dispersal; passively reducing the construction cost can at least ensure survival until favourable conditions.

Plant neighbour allelobiosis and allelopathy have been widely reported among species [60,61,62], but less is known about CIPs’ interactions. Various studies have demonstrated that the production of indirect defence traits is regulated by allelopathic interactions [63,64]. Allelopathy is broadly defined as a mechanism of inhibition, while allelobiosis is defined as a positive effect in chemical interactions [65], although their primary functions in interactions between CIPs differ [29]. Competition becomes more apparent when allelochemicals are removed from CIPs of IO. We hypothesise that allelobiosis between CIPs may mediate co-invasion, allowing for adjustments in biomass allocation and avoiding excessive resource investment in competitive and defensive behaviours, ultimately benefiting both itself and the population. Additionally, the soil conditioned by invasive plants can enhance the establishment of subsequent CIPs of IO. We hypothesise that their potential shared adaptations to the corresponding regional environment with CIPs [66]. After the removal of allelochemicals, the total biomass of CIPs of DO was consistently decreased by direct allelopathy and allelopathic legacy [67]. Additionally, the total biomass of IS of DOs was significantly greater than that of PS and instead became the predominant species. All species allocated more resources to ensure the advantages of basal diameter and coverage, which were pivotal factors in competition and nutrition acquisition [68]. Our hypothesis is that there is considerable allelopathy between PS and IS of DOs, making co-invasion more challenging.

**Figure 7:**
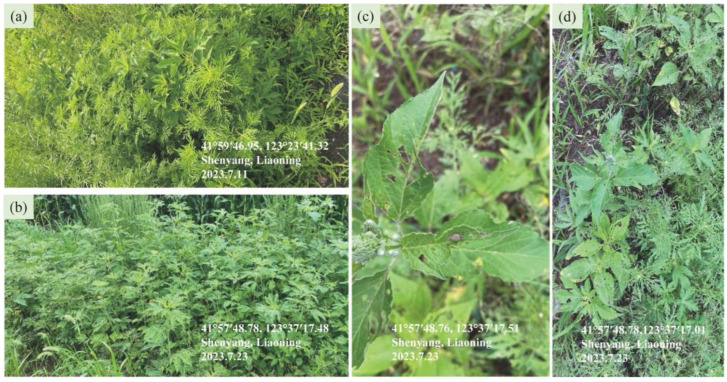
(**a**) Co-invasion of *Ambrosia trifida* and *Ambrosia artemisiifolia*. (**b**) *A. trifida* is fed by *Ophraella communa* when it invades alone. (**c**) *O. communa*. (**d**) *A. artemisiifolia* is preferentially fed when *A. trifida* and *A. artemisiifolia* co-invade.

Alien species may also be attacked by natural enemies of closely related species, thereby hindering their invasion [69]. However, our field observations reveal that when *A. trifida* and *A. artemisiifolia* co-invade, *Ophraella communa* prefers *A. artemisiifolia* (Figure 7). This preference allows *A. artemisiifolia* to share the burden of natural enemies with CIPs (*A. trifid*) and increases the likelihood of successful invasion. We believe that this is one of the reasons that CIPs of IO are more likely to co-invade the field.

The process of co-adaptation characterizes the co-evolution of ecologically closely and distantly related species, and the outcomes of this coevolutionary relatedness are contingent upon it. Any evolutionary advancement in one species can exert competitive pressure on other species, even under constant environmental conditions [70]. CIPs of IO are capable of ‘resource sharing’ (it takes one to know one) and can coexist in a habitat while competing with other species for resources [14,65,71]. CIPs of DO have apparent competition even under high nutrition levels. Allelopathic inhibition emerged as the dominant mechanism driving CIPs to compete when they co-invade, reflecting a strategic approach similar to ‘A bird in the hand is worth two in the bush’, thus preventing co-existence in a habitat. The co-invasion of CIPs varies due to differences in species origin, thus indicating that discussions on invasive plant dispersal should rely on more than just phylogeny [12,13].

## 5. Conclusions

This study conducted a controlled experiment to investigate the differential growth responses of invasive species to interaction and allelopathic legacy on CIPs of origin differences. CIPs of IO co-invade through allelobiosis and share resources in a habitat. On the contrary, CIPs of DO could regulate investment in competition and defence by allelopathy, thereby monopolizing habitat nutrition. Hence, when implementing biological control measures, it is crucial to prioritize monitoring and managing co-invasion and re-invasion by CIPs of IO existing invaders in invasive habitats, as these habitats exhibit heightened vulnerability to invasion.

## Data Availability

Data are available from the authors upon request.

## Supplementary Materials

The following supporting information can be downloaded at: https://www.mdpi.com/article/10.3390/plants13131807/s1, Table S1. Study species used in the experiment; Table S2. Species combinations used in Experiment 1; Table S3. Species combinations used in Experiment 2.
