# Supplementary material for: Co-Invasion of Congeneric Invasive Plants Adopts Different Strategies Depending on Their Origins
Source: Plants (Basel). 2024 Jun 30;13(13):1807. doi: 10.3390/plants13131807 (PMC11244186; doi:10.3390/plants13131807)
Supplement: Supplementary file 1 [file plants-13-01807-s001.zip › plants-3023606-supplementary.pdf]

Supporting information appendix

Table S1. Study species used in the experiment.

Species	Family	Genus	Origin	Is the origin the same?	Seed collection	Collection time	Co-ordinates
Ambrosia trifida	Asteraceae	*Ambrosia*	North America	-	Shenyang，Liaoning；	2021.10	123°33'54" 41°49'51.99"
Ambrosia artemisiifolia			North America	Yes	Dalian，Liaoning；	2021.10	123°14'23.61" 39°49'26.37"
Xanthium strumarium		*Xanthium*	North America	-	Shenyang，Liaoning；	2021.10	123°47'25.32" 41°45'59.01"
Xanthium Chinese			North America	Yes	Dalian，Liaoning；	2015.05	121°29'12.83" 38°58'6.42"
Tagetes erecta		*Tagetes*	North America	-	Dingxi，Gansu；	2021.10	104°5'32.08" 35°1'16.07"
Tagetes patula			North America	Yes	Suqian，Gansu；	2021.10	118°39'14.46" 34°11'54.20"

Tagetes minuta			Southern of South America	No	Yangquan，Shan	2022.03	113°45'38.07" 37°52'21.03"
Ipomoea nil	Convolvulaceae	*Ipomoea*	Tropical America	-	Dalian，Liaoning；	2021.10	122°8'42.64" 39°36'55.32"
Ipomoea purpurea			Tropical America	Yes	Shenyang，Liaoning；	2021.10	123°10'28.68" 42°12'23.14"
Solanum rostratum	Solanaceae	*Solanum*	North America	-	Chaoyang，Liaoning；	2018.10	119°34'52.41" 41°12'48.74"
Solanum sarrachoides			South America	No	Benxi，Liaoning；	2021.10	123°56'11.45" 41°20'15.12"
Bidens frondosa	Asteraceae	*Bidens*	North America	-	Chaoyang，Liaoning；	2021.10	119°4'57.99" 40°49'5.75"
Bidens bipinnata			Tropical America (Central America)	No	Fushun，Liaoning；	2021.10	123°57'48.99" 41°49'46.99"
Amaranthus retroflexus	Amaranthaceae	*Amaranthus*	North America	-	Fushun，Liaoning；	2021.10	123°57'24.00" 41°50'33.00"

Amaranthus lividus	Africa	No	Dalian，Liaoning；	2021.10	123°27'12.98" 39°55'5.77"
Amaranthus caudatus	South America	No	Shenyang，Liaoning；	2021.10	123°33'39.99" 41°49'40.00"

Co-invading invasive plant of congeneric indicates sites where both the alien and its related alien species were present. In case of *Ambrosia trifida*, the congeneric was *Ambrosia artemisiifolia.*

Table S2. Species combination used in the Experiment 1.

Origin	Treatments	Genus	Intraspecies competition	Interspecific competition
Identical origin	+N/-N-AC/+AC	*Ambrosia*	*A. trifida* + *A. trifida*	***A. trifida*** + *A. artemisiifolia*
			A. artemisiifolia + *A. artemisiifolia*	
		Xanthium	*X. strumarium* + *X. strumarium*	***X. strumarium*** + *X. Chinese*
			X. Chinese + *X. Chinese*	
		Tagetes	*T. erecta* + *T. erecta*	***T. erecta*** + *T. patula*
			T. patula + *T. patula*	
		Ipomoea	*I. nil* + *I. nil*	***I. nil*** + *I. purpurea*
			I. purpurea + *I. purpurea*	
Distinct origin	+N/-N-AC/+AC	*Bidens*	*B. frondosa* + *B. frondosa*	***B. frondosa*** + *B. bipinnata*
			B. bipinnata + *B. bipinnata*	
		Tagetes	*T. minuta* + *T. minuta*	***T. erecta*** + *T. minuta*
				T. patula + *T. minuta*
		Amaranthus	*A. retroflexus* + *A. retroflexus*	***A. retroflexus*** + *A. lividus*
			A. lividus + *A. lividus*	*A. retroflexus* + ***A. caudatus***
			A. caudatus + *A. caudatus*	***A. lividus*** + *A. caudatus*
		Solanum	*S. rostratum* + *S. rostratum*	***S. rostratum*** + *S. sarrachoides*
			S. sarrachoides + *S. sarrachoides*	

Bold fonts represent predominant species (PS) in interspecific competition (low-nutrition group -N-AC), and regular fonts represent inferior species (IS).

Table S3. Species combination used in the Experiment 2.

Origin	Genus	Group	Conditioning phase	Feedback phase
Identical origin	*Ambrosia*	CK	no plants	*A. artemisiifolia*
		-N	*A. trifida*	*A. artemisiifolia*
		+AC	*A. trifida*	*A. artemisiifolia* + activated carbon
	Xanthium	CK	no plants	*X. Chinese*
		-N	*X. strumarium*	*X. Chinese*
		+AC	*X. strumarium*	*X. Chinese* + activated carbon
	Tagetes	CK	no plants	*T. minuta*
		-N	*T. erecta*	*T. minuta*
		+AC	*T. erecta*	*T. minuta* + activated carbon
	Ipomoea	CK	no plants	*I. purpurea*
		-N	*I. nil*	*I. purpurea*
		+AC	*I. nil*	*I. purpurea* + activated carbon
		CK	no plants	*I. nil*
		-N	*I. purpurea*	*I. nil*
		+AC	*I. purpurea*	*I. nil* + activated carbon
Distinct origin	*Bidens*	CK	no plants	*B. bipinnata*
		-N	*B. frondosa*	*B. bipinnata*
		+AC	*B. frondosa*	*B. bipinnata* + activated carbon
	Tagetes	CK	no plants	*T. minuta*
		-N	*T. erecta*	*T. minuta*
		+AC	*T. erecta*	*T. minuta* + activated carbon
		CK	no plants	*T. minuta*
		-N	*T. patula*	*T. minuta*
		+AC	*T. patula*	*T. minuta* + activated carbon
	Amaranthus	CK	no plants	*A. lividus*
		-N	*A. retroflexu*	*A. lividus*
		+AC	*A. retroflexu*	*A. lividus* + activated carbon
		CK	no plants	*A. lividus*
		-N	*A. caudatus*	*A. lividus*

	+AC	*A. caudatus*	*A. lividus* + activated carbon
	CK	no plants	*S. sarrachoides*
Solanum	-N	*S. rostratum*	*S. sarrachoides*
	+AC	*S. rostratum*	*S. sarrachoides* + activated carbon
